# Expanding ligand-receptor interaction networks for axon guidance: Structural insights into signal crosstalk and specificity

**DOI:** 10.1016/j.conb.2025.102999

**Published:** 2025-03-20

**Authors:** Shaotong Zhu, Alexander Jaworski, Rob Meijers

**Affiliations:** 1 Institute for Protein Innovation, Boston, MA 02115, USA; 2 Department of Neuroscience, Brown University, Providence, RI 02912, USA; 3 Robert J. and Nancy D. Carney Institute for Brain Science, Providence, RI 02912, USA

## Abstract

Guidance of nascent axons to their targets is mediated by attractive and repulsive cues that activate receptors on the axonal growth cone. The number of ligand-receptor interactions implicated in axon pathfinding is still expanding, and large-scale cell-surface and extracellular protein interactome studies have revealed extensive crosstalk between signaling axes once thought to act independently. This raises the question how the apparent promiscuity of molecular interactions is compatible with specific signaling outcomes and effects on growth cone steering. Structural studies have provided insights into the modularity of binding interactions and shown the capacity of receptors to engage multiple ligands. Here, we review recent findings about the complexity of ligand-receptor interaction networks for axon guidance, and how structures of ligand-receptor complexes reveal mechanisms that may specify signaling output.

## Introduction

Assembly of neurons into information-processing networks is critical for nervous system function. Guidance of extending axons to their targets is a key step in neural circuit formation, and it is mediated by attractive and repulsive molecular cues that engage receptors on the axonal growth cone. In the 1990s, four major families of guidance cues – Netrins, Slits, Semaphorins, and Ephrins – and their cognate receptors were identified, and various cell adhesion molecules (CAMs) were also implicated in axonal navigation [1]. Discovery of novel guidance cue families has since slowed down; yet, identification of new molecular players continues to this day and has recently been spurred by large-scale protein-protein interaction (PPI) screens. These interactome studies have challenged long-standing notions about the exclusivity of ligand-receptor interactions and revealed extensive crosstalk between guidance molecules that were previously considered separate signaling axes. Meanwhile, structural and biophysical studies have helped decipher how guidance receptors change conformation and cluster in response to their ligands, and how this may instruct different signaling outcomes. In this review, we describe how our understanding of the expanding axon guidance PPI network is evolving, highlighting emerging principles of receptor complex organization while focusing on examples with clear implications for axon pathfinding.

## Extracellular interactome studies and evidence for extensive crosstalk between axon guidance molecules

Towards the end of the last century, biochemical purification of factors with *in vitro* axon guidance activity and genetic screens for mutations that cause axon pathfinding errors *in vivo* led to the identification of the major guidance cue families and their receptors. Subsequent work helped define roles of individual PPIs in specific pathfinding decisions and revealed that some membrane-anchored guidance cues can signal in reverse, blurring the lines between ligand and receptor identities. In recent years, high-throughput PPI screens have proven extremely powerful in discovering ligand-receptor pairs en masse, including many that might control neuronal wiring. A pioneering study of over 200 *D. melanogaster* immunoglobulin superfamily (IgSF), fibronectin type III (FnIII), and leucine-rich repeat (LRR) proteins identified a network of binding interactions between Dpr- and DIP-type IgSF members that are instrumental in neural circuit assembly [2,3], and between “Side” family guidance cues and “Beat” receptor proteins. Importantly, in both PPI networks, individual molecules interact with multiple, but not all, related binding partners [4,5]. How DIP-Dpr and Side-Beat interactions specify neuronal connectivity by promoting axon guidance, synaptic matching, or neuronal survival is still not fully understood [6].

Two recent studies have mapped the interactome of human IgSF members, each screening several hundred thousand pairwise binding events and finding hundreds of novel ligand-receptor pairs [7,8]. These screens identified a cornucopia of new binding partners for members of canonical guidance cue/receptor families. For instance, various new interactions were described for Netrin receptors of the DCC, Unc5, and DSCAM families. While the receptors DCC and Neogenin signal axon attraction downstream of Netrins and can also mediate the repulsive effects of Draxin and RGM family proteins, the three related DCC family members Punc, Nope, and Protogenin had remained orphan receptors. One interactome study implicated the secreted multi-domain protein WFIKKN2 as a novel binding partner for all five of these receptors [8]. In partial agreement, a screen of an extracellular protein library with the ectodomains of Punc, Nope, and Protogenin as baits identified WFIKKN2 as a ligand for these receptors, and WFIKKN2-Nope repulsive signaling was shown to control dorsal root ganglion peripheral sensory axon guidance in mice [9]. DCC and Neogenin failed to bind WFIKKN2 in this study, revealing strict functional specialization among members of the mouse DCC receptor family. For the Unc5 family, the other IgSF interactome study explored the full extent of interactions between Unc5 and FLRT family members [7], which mediate repulsion, confirming earlier studies [10–12] and identifying additional binding events in this network. This study also discovered that Unc5 receptors bind to Glypican-3 and Sema7A [7], and binding of Glypican-3 to Unc5d was recently shown to mediate repulsion and promote cortical neuron migration in mice [13]. This establishes a clear chemotropic activity for this PPI, but whether Glypican-Unc5 complexes regulate axon pathfinding remains to be determined. *Drosophila* DSCAM was previously shown to form a complex with the receptor tyrosine phosphatase RPTP69D, which inhibits DSCAM function in axon branching downstream of Slit [14]. Several RPTP family members were found to bind the DSCAM ectodomain in one of the human IgSF interactome screens, supporting relevance of this inhibitory interaction for human biology [8].

Very recently, an investigation of over 72,000 possible pairwise interactions between *C. elegans* receptor ectodomains and secreted proteins was carried out [15]. This study covered a wider range of molecules beyond IgSF, FnIII, and LRR proteins, identifying entirely novel binding partners for the Netrin receptors UNC-5 and UNC-40 (the worm DCC ortholog) and various other guidance molecules. It also uncovered multiple points of extracellular crosstalk between the major axon guidance signaling axes: the Robo family member SAX-3 (a receptor for Slit proteins) binds the Ephrin EFN-4 and the Plexin PLX-1 (a Semaphorin receptor), and EFN-4 binds the Semaphorin MAB-20.

Functions in axon guidance for most of the newly discovered ligand-receptor complexes remain to be established experimentally, but, so far, *in vivo* investigation of the PPIs discovered in these large-scale screens confirms their relevance [9,13,14], underscoring the value of unbiased interactome mapping. It will also be important to systematically compare extracellular interactomes across species, as the expansion of gene families in higher organisms is expected to allow greater functional specialization of proteins. The extensive crosstalk between canonical axon guidance pathways observed in *C. elegans* might therefore represent a specialized case, as it was not observed in an earlier study on the interactome of floor plate-derived proteins in D. rerio [7] or the human IgSF interactome screens [7,8].

## Other novel protein–protein interactions for axon guidance

Ever since the discovery of the first guidance molecules, targeted genetic investigation of specific axon guidance decisions or protein families implicated in neural circuit assembly has gradually expanded the known molecular toolkit for axon pathfinding by identifying additional cues and receptors. Among recently discovered extracellular PPIs with functional relevance for axon guidance is binding of the *Drosophila* Semaphorin Sema1a to the Wnt receptor Off-track (OTK) [16], which provides a molecular mechanism for OTK’s apparent function in axon repulsion and its physical association and genetic interaction with the Semaphorin receptor Plexin A. In developing mouse midbrain dopaminergic neurons, the CAM Alcam was implicated in modulating Semaphorin signaling through trans interactions with the Semaphorin co-receptor Neuropilin1; additionally, Alcam promotes axon extension by trans binding to the closely related CAMs L1 and Chl1 [17]. Importance of these Alcam interactions for axon development *in vivo* has not been fully explored.

While transmembrane proteins of the Teneurin family had previously been implicated in synapse formation through heterophilic binding to Latrophilin family adhesion GPCRs, studies in the developing mouse hippocampus have now identified bona fide axon guidance functions for Ten-3 and Lphn2 [18–20]. Interestingly, Ten-3 can function as both a ligand and receptor for axon guidance, eliciting attraction through homophilic Ten3-Ten3 binding or repulsion through heterophilic Ten3-Lphn2 or Lphn2-Ten3 binding. BAI family adhesion GPCRs promote synapse formation by interacting with Neuroligins and C1q-like proteins. BAI1 and BAI3 were also found to bind RTN4R/NgR, known as an inhibitory receptor in axon regeneration, and signaling from glial BAIs to neuronal RTN4R promotes axon growth in cultured human neurons [21], although the *in vivo* role of this interaction remains to be explored in depth. The adhesion GPCR CELSR3, which is an ortholog of the *D. melanogaster* planar cell polarity protein Flamingo and has been implicated in Wnt-dependent axon guidance, was recently found to bind Dystroglycan, and this PPI appears to be critical for mouse spinal commissural axon guidance [22]. While it is still unclear whether the Dystroglycan-CELSR3 complex produces signaling output in the absence of other CELSR3 ligands and is instructive for axon pathfinding, this study adds to the growing list of Dystroglycan binding partners, which also includes Slit family axon repellants.

The *D. melanogaster* RPTP Lar guides various populations of axons, and some, but not all, aspects of these functions can be explained by interactions with the Lar binding partners Syndecan and Dally-like, which are heparan sulfate proteoglycans. Recent work shows that Lar also functions as receptor for the Nephrin family CAM Sticks and Stones (Sns), which itself is known to bind other, closely related CAMs, and this interaction is required for photoreceptor axon targeting and guidance of mushroom body Kenyon cell axons [23]. Two mammalian orthologs of Lar, PTPδ and PTPσ, were recently identified as receptors for collagen XXV, a transmembrane collagen [24]. This PPI is critical for intramuscular motor axon growth and muscle innervation in mice, and it is disrupted in certain forms of Congenital Cranial Dysinnervation Disorder.

Given the plethora of novel extracellular PPIs that were recently found to be important for axon pathfinding, it can be concluded that the full repertoire of ligand-receptor interactions for axon guidance remains to be delineated. Moreover, the growing list of interactions between members of the known axon guidance ligand-receptor network implies a dizzying array of molecular binding events that raises questions about how individual receptors process such varied inputs. What is the capacity of a receptor to bind multiple partners at the same time, and how does ligand competition and multiplicity affect receptor signaling?

## Structural insights into receptor binding to multiple partners

**Figure 1:**
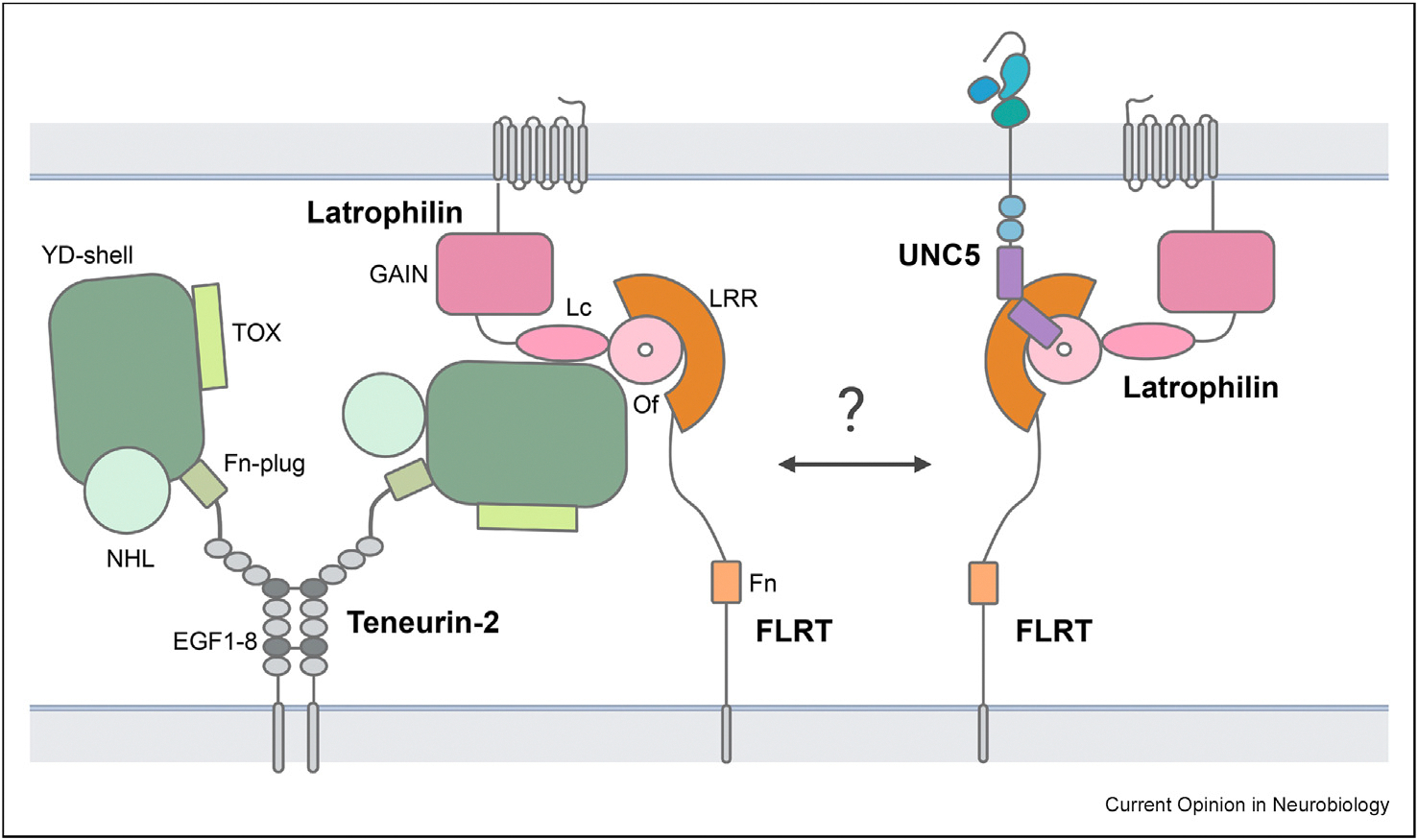
Structure-based cluster formations of the Teneurin-Latrophilin hub. Schematic of the Teneurin receptor hub, consisting of a Teneurin-2 dimer engaging Latrophilin on an opposite cell (in trans) and FLRT on the same cell (in cis). The formation of a supercomplex involving Unc5 with Teneurin is yet to be determined, so a Latrophilin-FLRT-Unc5 complex is drawn separately. Individual domains are denoted for Teneurin-2 for the toxin-like domain (TOX), tyrosine/aspartate repeats domain (YD shell), fibronectin plug domain (Fn-plug), NCL-1, HT2A and Lin-41 domain (NHL) and epidermal growth factor-like (EGF) domains 1 to 8 (EGF1–8). For Latrophilin, the GAIN, Olfactomedin (Of) and Lectin (Lc) domains are depicted, and for FLRT the leucine-rich repeat (LRR) and Fibronectin domain (Fn).

Structural biology has helped elucidate the ability of guidance receptors to engage with multiple different ligands, identifying various receptor clustering patterns that suggest specific signaling outputs. One exciting area where structural studies synergize with functional interactome analysis involves the Teneurin/Latrophilin hub (Figure 1). Initial studies mapped the interactions between Teneurin and Latrophilin family members to the lectin domain of Latrophilin and the YD shell of Teneurin, with 1:1 stoichiometry [25–28]. Latrophilin also binds FLRTs, and a complex between Latrophilin, FLRT, and Unc5 is formed with 2:1:1 stoichiometry [10,29]. Whether Latrophilin bridges Teneurins and FLRTs to form a supercomplex remains unclear. It is interesting that Unc5 is recruited into this complex, as it was originally identified as a repulsive Netrin receptor; this provides a striking example of an axon guidance receptor that engages several signaling hubs. As mentioned, Unc5 also forms a complex with Glypican-3, as was revealed by interactome studies [7]. A structure of Glypican-3 bound to Unc5d revealed a compact complex with 4:4 stoichiometry that could promote either cis or trans signaling, and disruption of this complex by nanobodies supported a crucial role in neuronal and neuroblastoma migration [13]. It is noteworthy that presence of Unc5 in three distinct receptor complexes consistently contributes to repulsive signaling. Recently, it was shown that Teneurin alternative splicing may affect homodimerization and complex formation, and some variants may exclude certain heterophilic PPIs [28,30,18]. There is still much to explore around the functional role of alternative splicing of Teneurins in the formation of larger complexes involving FLRT and Latrophilin family members and in cis and trans interactions [31].

**Figure 2:**
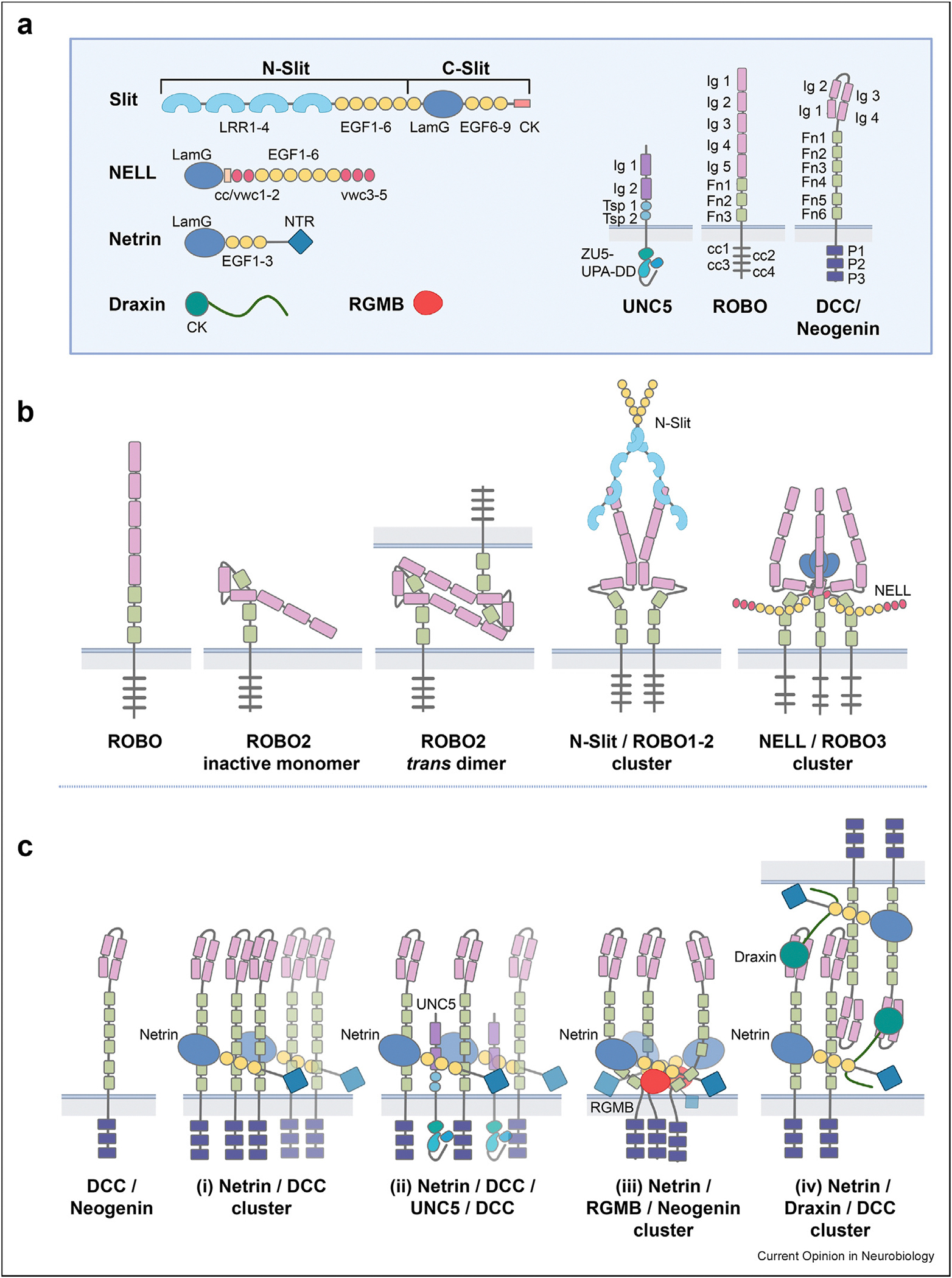
Structure-based cluster formations of the Slit-Robo and Netrin-DCC ligand-receptor hubs. a) Individual domains for ligands and receptors involved in the Slit-ROBO and Netrin-DCC hubs. Receptors contain Ig (immunoglobulin-like), Fn (fibronectin) and Tsp (Thrombospondin) domains. Slit consists of four leucine-rich repeats (LRR1–4), six EGF domains (EGF1–6), a laminin-like domain (LamG), three EGF domains (EGF6–9) and a C-terminal cysteine knot (CK), and it is cleaved into an N-Slit and C-Slit module [59]. NELL consists of a laminin-like domain (LamG), followed by a coiled coil (cc) and two Willebrand factor type C (vwc) domains (cc/wvc1–2), six EGF domains (EGF1–6) and three Willebrand factor type C domains (VWC3–5). Netrin consists of a laminin-like domain (LamG), three EGF domains (EGF1–3) and a netrin-like domain (NTR). Draxin contains a cysteine knot domain (CK). b) Schematic of the Robo hub, showing an inactive monomeric Robo2 receptor and trans dimer, and ligand-induced clustering by N-Slit as a dimer of dimers for Robo1 and Robo2, as well as by a NELL2 trimer core forming heterodimers with Robo3. c) Schematic of the DCC/Neogenin hub. Both DCC and Neogenin share the same domain structure and interactions with Netrin, but the RGM ligand only engages Neogenin while the Draxin ligand only engages DCC. For the Netrin/DCC cluster (i), each Netrin molecule can cluster three DCC receptors which each can be replaced by Neogenin. A daisy chain of Netrin/DCC clusters can be constructed due to the arrangement of the DCC binding sites. (ii) Unc5 replaces DCC or Neogenin at the EGF-2 domain of Netrin. (iii) RGM stabilizes a single 3:3:3 Netrin-Neogenin complex, while (iv) Draxin displaces DCC at the EGF-3 domain of Netrin, disrupting the daisy chain of Netrin/DCC clusters, and at the same time binds to the N-terminal Ig domains of an opposing DCC receptor in trans. The figure was produced with Biorender.

Mammalian Robo receptors form monomers or multimers, and they can mediate signaling from Slit and NELL ligands (Figure 2a and b). It was shown that Robo1 and Robo2 assume an inactive conformation where the tip of the receptor is folded onto the base, masking the Ig4 domain crucial in signaling [32]. Robos in this inactive conformation form dimers in trans and possibly in cis [32,33]. Slits, which function as dimers, activate these receptors through a mechanism that remains unclear, potentially involving the formation of an activated Robo dimer mediated by the Ig4 domain [33]. NELL2 binds a cryptic site on Robo1/2 that becomes available when the receptor ectodomain changes conformation from a closed, hairpin structure to an open, elongated state [34,35]. NELL2 binds Robo3 without the need for a conformational shift, as Robo3 appears to predominantly assume the open conformation [34]. NELL2 forms a trimer and likely signals by triggering formation of larger Robo receptor clusters. The binding sites for Slits and NELLs on Robos are distinct, as Slits bind to the most N-terminal Ig domain, away from the cell membrane, while NELLs interact with the first of three membrane-proximal FnIII domains. DSCAM is another axon guidance receptor that binds Slit at the receptor N-terminus and a second ligand, Netrin, closer to the membrane [36]. This suggests that localization of these cues on their receptors as membrane-proximal or membrane-distal cues may relate to their function in receptor clustering.

The understanding of the Netrin signaling interactome has also benefited from extensive structural analysis (Figure 2c). Initial structures of Netrin in complex with DCC and Neogenin established three receptor binding sites on the laminin and EGF domains of Netrin [37,38], so that Netrin induces formation of DCC lattices. Structural studies on additional ligands modulating Netrin signaling revealed competing and complementary binding modes. Draxin was shown to contain two adjacent binding sites, namely a linear peptide that binds Netrin with high affinity and competes with one of the DCC binding sites, and a cysteine knot domain that binds to the N-terminal horseshoe-shaped Ig domains on the tip of DCC. This rearrangement of the Netrin-DCC interface by Draxin disrupts the Netrin-DCC lattice on the same cell surface [39] and promotes trans cell-cell interactions. Indeed, Draxin has been implicated in axonal fasciculation which is supported by axon-axon adhesion. In contrast, a ternary complex structure involving Netrin, Neogenin, and the repulsive guidance cue RGM revealed a modular system that permits concomitant binding of Netrin and RGM to Neogenin into a supramolecular complex with 3:3:3 stoichiometry [40]. Functional experiments in the same study showed that RGM association with Netrin/Neogenin silences receptor signaling. Draxin and RGM therefore seem to have contrasting roles in altering Netrin signaling, as reflected in a reorganization of the ligand-receptor clusters. Draxin promotes trans receptor interactions to facilitate fasciculation, whereas RGM may alter cis DCC interactions to attenuate Netrin signaling. These alternate cluster formations may serve as an inspiration in the study of many other single-pass transmembrane receptor-ligand systems.

## Glycosaminoglycans affecting receptor complex formation and signaling

Another important principle that has come to light through structural studies of these ligand-receptor pairs is the role that sugar-based polymers play in controlling complex formation, receptor clustering, and signaling. One of the hypotheses inferred from the early Netrin/DCC studies was that Unc5 would compete for one of the DCC binding sites, indicating competition amongst Neogenin, DCC, Unc5 and Draxin. The structure suggested that an arginine-rich region on the Netrin EGF-2 domain would require glycosaminoglycan (GAG) molecules for receptor binding [37]. A study using domain deletions further supported Unc5b binding to the EGF-2 domain of chicken Netrin [41]. In a followup study, it was suggested that the EGF-2 site was also involved in Netrin oligomerization through interactions with heparan sulfate chains [42]. In contrast, a study on *C. elegans* and human Netrin revealed that heparan sulfate plays a central role in strengthening the interaction between Unc5 and Netrin at the EGF-2 domain, while simultaneously weakening binding between DCC and Netrin at the same site [43]. Heparan sulfate therefore seems to play a dual role: 1) as modulator of Netrin-receptor interactions and 2) as aggregator of Netrin in the extracellular matrix. Incidentally, an antibody targeting the region of the human Netrin EGF-2 domain that interferes with Netrin-Unc5b binding has shown promise to halt Netrin-mediated tumor progression in endometrial carcinoma [44,45]. It remains to be seen if the heparan sulfate involved in Netrin signaling is linked to a particular proteoglycan, or if it originates from free-floating fragments. Interestingly, a peculiar glycan-glycan interaction lies at the core of the complex between Unc5d and Glypican-3 [13]. A mannosylated tyrosine on Unc5d interacts with an N-glycan on Glypican-3 at the core of the binding interface. A similar glycan-specific interaction has been shown at the center of the BAI adhesion GPCR complex with RTN4R [21]. GAGs also play a role in Semaphorin signaling, where a switch from attractive to repulsive guidance activity of the transmembrane Semaphorin Sema5A is driven by this cue’s binding to chondroitin sulfate [46]. While stoichiometry of the Semaphorin-Plexin-Neuropilin complexes has been structurally verified [47–49], novel insights were obtained into how Semaphorin-GAG interactions regulate higher-order clustering of these complexes. Sema5A oligomerization depends on heparan sulfate-containing proteoglycans [50], and secreted *Drosophila* Semaphorins form GAG-induced dimers [16,47] that associate with cell-bound GAGs away from the Plexin receptor binding site [51]. These studies further indicate that GAGs can play important roles in modulating ligand-receptor interactions [52]. GAGs may capture ligands to increase their local concentration, limit ligand diffusion, concatenate ligand-receptor clusters together, and act as molecular switches to promote or inhibit specific ligand-receptor interactions.

## Receptor cluster reorganization as switch from cell-autonomous to cell-cell signaling

**Figure 3:**
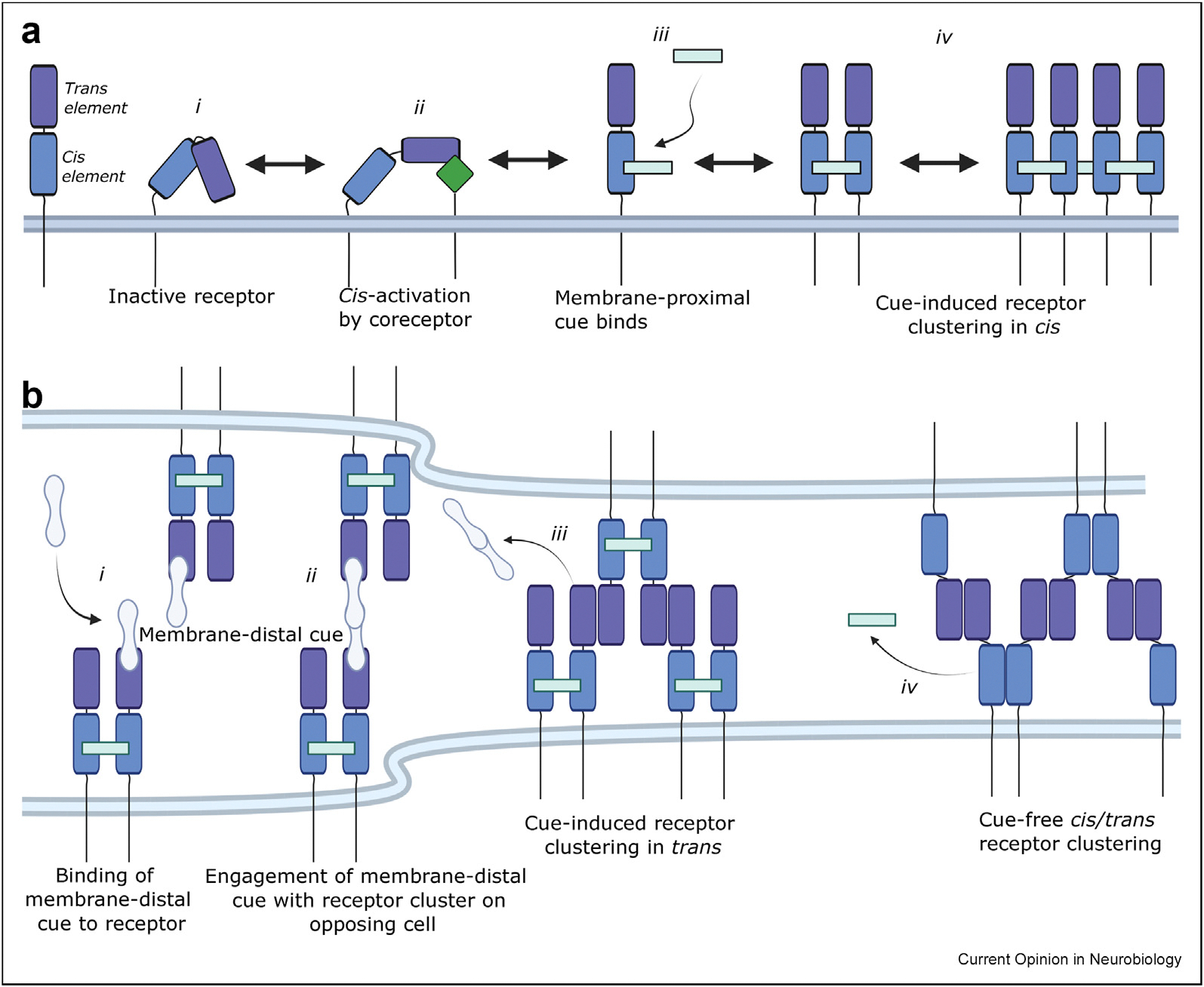
Schematic of tentative model for single-pass transmembrane receptor activation and ligand engagement leading to cell-autonomous and cell-cell cluster formation. a) Schematic of a single-pass transmembrane receptor containing a membrane-distal *trans* element (dark blue) and a membrane-proximal *cis* element (light blue). The inactive receptor (i) is activated by a co-receptor (ii) or directly by a ligand that acts as a membrane proximal cue (iii), leading to receptor clustering in *cis* (iv). b) Schematic of ligand-induced clustering of receptors across two opposing cell membranes. Binding of a ligand to the trans element of the receptor as a membrane-distal cue (i) leads to engagement of receptors positioned on opposing cells (ii). When the ligand acting as a membrane-distal cue diffuses out, the receptor clusters in *trans* (iii), stabilizing the adhesive lattice of receptors between cells. With the adhesive trans receptor lattice in place, the membrane proximal cue diffuses away and direct *cis* interactions between receptors on the same cell are formed (iv). Examples for membrane-proximal cues are Netrin for DCC and DSCAM receptors, and NELL for Robo receptors. Examples for membrane-distal cues are Draxin for the DCC receptor, and Slits for Robo and DSCAM receptors.

Axonal sampling of the cellular environment involves interactions on the growth cone itself (cis interactions) as well as interactions with surrounding tissues and neighboring axons (trans interactions). The ligand-receptor systems discussed here can toggle between cis and trans interactions, and thereby switch from cell-autonomous signaling to cell-cell engagement. From structural studies, a concept emerges where guidance receptors assume different conformations to engage with their ligands (Figure 3a), requiring conformational shifts that activate the receptors to unmask guidance cue binding sites. Conversely, guidance cues may constrain the conformations of the otherwise flexible IgSF receptors, where some cues (e.g. Netrin, NELL) bind receptors close to the cell membrane, inducing or disrupting receptor cis interactions. Other cues (e.g. Slit, Draxin) bind the membrane-distal part of receptors, thereby modulating trans interactions (Figure 3b). This is a variation on the receptor zipper model, which has been extensively characterized for Protocadherins [53]. There, cis interactions between Protocadherins occur close to the membrane and trans binding occurs at the membrane-distal domains of these rod-like receptors [54]. Electron tomograms of reconstituted, membrane-embedded Protocadherin [55] and DSCAM [56] show highly periodic clusters engaged in cis and trans interactions. It remains to be seen how ligands and other co-receptors may disrupt or promote these zipper arrangements.

## Conclusion and perspective

The iconic view that axon pathfinding is regulated by a few families of canonical guidance cues and highly specific receptors has recently been challenged, as the complexity of interactions between ligands and receptors from different families has come to light. Largescale interactome studies have started to uncover the expanse of cross-family interactions, and structural studies have revealed how receptors can accommodate a multitude of co-receptor and ligand interactions. Some general patterns can be discerned that contribute to a more nuanced picture of ligand-receptor signaling in axon guidance.

- Most ligand-receptor pairs interact with other binding partners.
- These partners can modulate signaling of the ligand-receptor pair through changes in ligand engagement, receptor clustering, and facilitation of cell-autonomous or cell-cell interactions; or they induce signaling by themselves.
- Only a small subset of the potential interactome is likely to be active at any particular point of an axon’s trajectory. Receptors are selectively silenced by ligands, GAGs, or co-receptors, as shown for the Netrin receptors DCC and Unc5.

Selective and temporally restricted use of specific, highly modular, and often cross-regulatory binding interactions may allow the axon guidance interactome to rapidly adapt to new binding partners or extracellular matrix modifications encountered along the axonal path, supporting efficient growth cone steering. From an evolutionary perspective, the emergence of more complex nervous systems in higher vertebrates coincided with the expansion of gene families encoding axon pathfinding molecules [57]. This allows more flexible spatiotemporal regulation of gene expression while enabling proteins to specialize, shedding redundant functions of their multifunctional ancestors, as exemplified in the mammalian Robo family, where high-affinity Slit and NELL receptors are largely non-overlapping [34]. Whether guidance cue crosstalk is an evolutionarily ancient or more recent feature of the neuronal wiring interactome, and the rate at which molecular interactions are added to or eliminated from this PPI network over the course of phylogenesis, remains to be determined.

The expanded extracellular interactome for axon guidance is a general blueprint for potential ligand-receptor systems engaging with each other, but its functional relevance hinges on cellular context. Single-cell and spatial transcriptomics have opened the door for inferring cell-cell communication axes based on the expression patterns of ligands and receptors in cells that are in physical proximity *in vivo*. Various computational tools leverage information about extracellular PPIs to predict “sender” and “receiver” cells, with respect to known signaling pathways, from gene expression data [58]. Continued expansion of the ligand-receptor interactomes for axon guidance will increase the power of these approaches in generating testable hypotheses about the functions of axon guidance molecules *in vivo*.

Structural studies to date have offered intriguing static snapshots of various receptor states and cluster arrangements. However, much is still to be learned about receptor dynamics, including conformational changes and interactions. Detailed structural understanding of the axon guidance interactome will benefit from a more holistic approach combining whole-cell imaging techniques with high-throughput annotation of structural components by artificial intelligence-powered machine learning algorithms. Technical advances in electron tomography and light microscopy, combined with increasing availability of antibodies and nanobodies specific for individual receptors and conformational states at cell–cell interfaces create exciting opportunities to visualize complex ligand-receptor interactomes in the context of a moving growth cone.

## Data Availability

No data was used for the research described in the article.

## References

[1] Tessier-Lavigne M, Goodman CS: The molecular biology of axon guidance. Science 1996, 274:1123–1133. 8895455 10.1126/science.274.5290.1123

[2] Özkan E, Carrillo RA, Eastman CL, Weiszmann R, Waghray D, Johnson KG, Zinn K, Celniker SE, Garcia KC: An extracellular interactome of immunoglobulin and LRR proteins reveals receptor-ligand networks. Cell 2013, 154:228–239. 23827685 10.1016/j.cell.2013.06.006 PMC3756661

[3] Cheng S, Ashley J, Kurleto JD, Lobb-Rabe M, Park YJ, Carrillo RA, Özkan E: Molecular basis of synaptic specificity by immunoglobulin superfamily receptors in Drosophila. Elife 2019, 8, e41028. 30688651 10.7554/eLife.41028 PMC6374074

[4] Li H, Watson A, Olechwier A, Anaya M, Sorooshyari SK, Harnett DP, Lee H-K (Peter), Vielmetter J, Fares MA, Garcia KC, : Deconstruction of the beaten Path-Sidestep interaction network provides insights into neuromuscular system development. Elife 2017, 6, e28111. 28829740 10.7554/eLife.28111 PMC5578738

[5] Cosmanescu F, Katsamba PS, Sergeeva AP, Ahlsen G, Patel SD, Brewer JJ, Tan L, Xu S, Xiao Q, Nagarkar-Jaiswal S, : Neuron-subtype-specific expression, interaction affinities, and specificity determinants of DIP/Dpr cell recognition proteins. Neuron 2018, 100:1385–1400.e6. 30467080 10.1016/j.neuron.2018.10.046 PMC6309224

[6] Wolterhoff N, Hiesinger PR: Synaptic promiscuity in brain development. Curr Biol 2024, 34:R102–R116. 38320473 10.1016/j.cub.2023.12.037 PMC10849093

[7] Verschueren E, Husain B, Yuen K, Sun Y, Paduchuri S, Senbabaoglu Y, Lehoux I, Arena TA, Wilson B, Lianoglou S, : The immunoglobulin superfamily receptome defines cancer-relevant networks associated with clinical outcome. Cell 2020, 182:329–344.e19. 32589946 10.1016/j.cell.2020.06.007

[8] Wojtowicz WM, Vielmetter J, Fernandes RA, Siepe DH, Eastman CL, Chisholm GB, Cox S, Klock H, Anderson PW, Rue SM, : A human IgSF cell-surface interactome reveals a complex network of protein-protein interactions. Cell 2020, 182:1027–1043.e17. 32822567 10.1016/j.cell.2020.07.025 PMC7440162

[9] Nickerson KR, Tom I, Cortés E, Abolafia JR, Özkan E, Gonzalez LC, Jaworski A: WFIKKN2 is a bifunctional axon guidance cue that signals through divergent DCC family receptors. 2023, 10.1101/2023.06.15.544950.

[10] Jackson VA, Mehmood S, Chavent M, Roversi P, Carrasquero M, Del Toro D, Seyit-Bremer G, Ranaivoson FM, Comoletti D, Sansom MSP, : Super-complexes of adhesion GPCRs and neural guidance receptors. Nat Commun 2016, 7, 11184. 27091502 10.1038/ncomms11184 PMC4838878

[11] Lu YC, Nazarko OV, Sando R, Salzman GS, Li N-S, Südhof TC, Araç D: Structural basis of latrophilin-FLRT-UNC5 interaction in cell adhesion. Structure 2015, 23:1678–1691. 26235030 10.1016/j.str.2015.06.024 PMC4851429

[12] Gao X, Metzger U, Panza P, Mahalwar P, Alsheimer S, Geiger H, Maischein H-M, Levesque MP, Templin M, Söllner C: A floor-plate extracellular protein-protein interaction screen identifies Draxin as a secreted netrin-1 antagonist. Cell Rep 2015, 12:694–708. 26190107 10.1016/j.celrep.2015.06.047

[13] Akkermans O, Delloye-Bourgeois C, Peregrina C, Carrasquero-Ordaz M, Kokolaki M, Berbeira-Santana M, Chavent M, Reynaud F, Raj R, Agirre J, : GPC3-Unc5 receptor complex structure and role in cell migration. Cell 2022, 185:3931–3949.e26. 36240740 10.1016/j.cell.2022.09.025 PMC9596381

[14] Dascenco D, Erfurth M-L, Izadifar A, Song M, Sachse S, Bortnick R, Urwyler O, Petrovic M, Ayaz D, He H, : Slit and receptor tyrosine phosphatase 69D confer spatial specificity to axon branching via Dscam1. Cell 2015, 162:1140–1154. 26317474 10.1016/j.cell.2015.08.003 PMC4699798

[15] Nawrocka WI, Cheng S, Hao B, Rosen MC, Cortés E, Baltrusaitis EE, Aziz Z, Kovács IA, Özkan E: Nematode extracellular protein interactome expands connections between signaling pathways. bioRxiv 2024, 10.1101/2024.07.08.602367. PMC13174244 41653916

[16] Rozbesky D, Monistrol J, Jain V, Hillier J, Padilla-Parra S, Jones EY: Drosophila OTK is a glycosaminoglycan-binding protein with high conformational flexibility. Structure 2020, 28:507–515.e5. 32187531 10.1016/j.str.2020.02.008 PMC7203548

[17] Bye CR, Rytova V, Alsanie WF, Parish CL, Thompson LH: Axonal growth of midbrain dopamine neurons is modulated by the cell adhesion molecule ALCAM through trans-heterophilic interaction with L1Cam, ChL1 and Semaphorin. J Neurosci 2019, 10.1523/JNEUROSCI.0278-19.2019. PMC6703882 31300520

[18] Berns DS, DeNardo LA, Pederick DT, Luo L: Teneurin-3 controls topographic circuit assembly in the hippocampus. Nature 2018, 554:328–333. 29414938 10.1038/nature25463 PMC7282895

[19] Donohue JD, Amidon RF, Murphy TR, Wong AJ, Liu ED, Saab L, King AJ, Pae H, Ajayi MT, Anderson GR: Parahippocampal latrophilin-2 (ADGRL2) expression controls topographical presubiculum to entorhinal cortex circuit connectivity. Cell Rep 2021, 37, 110031. 34818557 10.1016/j.celrep.2021.110031

[20] Pederick DT, Lui JH, Gingrich EC, Xu C, Wagner MJ, Liu Y, He Z, Quake SR, Luo L: Reciprocal repulsions instruct the precise assembly of parallel hippocampal networks. Science 2021, 372:1068–1073. 34083484 10.1126/science.abg1774 PMC8830376

[21] Wang J, Miao Y, Wicklein R, Sun Z, Wang J, Jude KM, Fernandes RA, Merrill SA, Wernig M, Garcia KC, : RTN4/NoGo-receptor binding to Bai adhesion-GPCRs regulates neuronal development. Cell 2021, 184:5869–5885.e25. 34758294 10.1016/j.cell.2021.10.016 PMC8620742

[22] Lindenmaier LB, Parmentier N, Guo C, Tissir F, Wright KM: Dystroglycan is a scaffold for extracellular axon guidance decisions. Elife 2019, 8, e42143. 30758284 10.7554/eLife.42143 PMC6395066

[23] Bali N, Lee H-K (Peter), Zinn K: Sticks and Stones, a conserved cell surface ligand for the Type IIa RPTP Lar, regulates neural circuit wiring in Drosophila. Elife 2022, 11, e71469. 35356892 10.7554/eLife.71469 PMC9000958

[24] Munezane H, Oizumi H, Wakabayashi T, Nishio S, Hirasawa T, Sato T, Harada A, Yoshida T, Eguchi T, Yamanashi Y, : Roles of collagen XXV and its putative receptors ptps/δ in intramuscular motor innervation and congenital cranial dysinnervation disorder. Cell Rep 2019, 29:4362–4376.e6. 31875546 10.1016/j.celrep.2019.11.112

[25] Sando R, Jiang X, Südhof TC: Latrophilin GPCRs direct synapse specificity by coincident binding of FLRTs and teneurins. Science 2019, 363, eaav7969. 30792275 10.1126/science.aav7969 PMC6636343

[26] Zhang X, Lin P-Y, Liakath-Ali K, Südhof TC: Teneurins assemble into presynaptic nanoclusters that promote synapse formation via postsynaptic non-teneurin ligands. Nat Commun 2022, 13:2297. 35484136 10.1038/s41467-022-29751-1 PMC9050732

[27] del Toro D, Carrasquero-Ordaz MA, Chu A, Ruff T, Shahin M, Jackson VA, Chavent M, Berbeira-Santana M, Seyit-Bremer G, Brignani S, : Structural basis of teneurin-latrophilin interaction in repulsive guidance of migrating neurons. Cell 2020, 180:323–339.e19. 31928845 10.1016/j.cell.2019.12.014 PMC6978801

[28] Li J, Xie Y, Cornelius S, Jiang X, Sando R, Kordon SP, Pan M, Leon K, Südhof TC, Zhao M, : Alternative splicing controls teneurin-latrophilin interaction and synapse specificity by a shape-shifting mechanism. Nat Commun 2020, 11:2140. 32358586 10.1038/s41467-020-16029-7 PMC7195488

[29] Jackson VA, del Toro D, Carrasquero M, Roversi P, Harlos K, Klein R, Seiradake E: Structural basis of latrophilin-FLRT interaction. Structure 2015, 23:774–781. 25728924 10.1016/j.str.2015.01.013 PMC4396693

[30] Gogou C, Beugelink JW, Frias CP, Kresik L, Jaroszynska N, Drescher U, Janssen BJC, Hindges R, Meijer DH: Alternative splicing controls teneurin-3 compact dimer formation for neuronal recognition. Nat Commun 2024, 15:3648. 38684645 10.1038/s41467-024-47763-x PMC11058771

[31] Meijer DH, Frias CP, Beugelink JW, Deurloo YN, Janssen BJC: Teneurin4 dimer structures reveal a calcium-stabilized compact conformation supporting homomeric trans-interactions. EMBO J 2022, 41, e107505. 35099835 10.15252/embj.2020107505 PMC9058538

[32] Barak R, Yom-Tov G, Guez-Haddad J, Gasri-Plotnitsky L, Maimon R, Cohen-Berkman M, McCarthy AA, Perlson E, Henis-Korenblit S, Isupov MN, : Structural principles in Robo activation and auto-inhibition. Cell 2019, 177:272–285.e16. 30853216 10.1016/j.cell.2019.02.004

[33] Aleksandrova N, Gutsche I, Kandiah E, Avilov SV, Petoukhov MV, Seiradake E, McCarthy AA: Robo1 forms a compact dimer-of-dimers assembly. Structure 2018, 26:320–328.e4. 29307485 10.1016/j.str.2017.12.003 PMC5807052

[34] Pak JS, DeLoughery ZJ, Wang J, Acharya N, Park Y, Jaworski A, Özkan E: NELL2-Robo3 complex structure reveals mechanisms of receptor activation for axon guidance. Nat Commun 2020, 11:1489. 32198364 10.1038/s41467-020-15211-1 PMC7083938

[35] Miyaguchi M, Nakanishi Y, Maturana AD, Mizutani K, Niimi T: Conformational change of the hairpin-like-structured Robo2 ectodomain allows NELL1/2 binding. J Mol Biol 2022, 434, 167777. 35940226 10.1016/j.jmb.2022.167777

[36] Alavi M, Song M, King GLA, Gillis T, Propst R, Lamanuzzi M, Bousum A, Miller A, Allen R, Kidd T: Dscam1 forms a complex with Robo1 and the N-terminal fragment of Slit to promote the growth of longitudinal axons. PLoS Biol 2016, 14, e1002560. 27654876 10.1371/journal.pbio.1002560 PMC5031454

[37] Finci LI, Krüger N, Sun X, Zhang J, Chegkazi M, Wu Y, Schenk G, Mertens HDT, Svergun DI, Zhang Y, : The crystal structure of netrin-1 in complex with DCC reveals the bifunctionality of netrin-1 as a guidance cue. Neuron 2014, 83:839–849. 25123307 10.1016/j.neuron.2014.07.010 PMC4412161

[38] Xu K, Wu Z, Renier N, Antipenko A, Tzvetkova-Robev D, Xu Y, Minchenko M, Nardi-Dei V, Rajashankar KR, Himanen J, : Structures of netrin-1 bound to two receptors provide insight into its axon guidance mechanism. Science 2014, 344: 1275–1279. 24876346 10.1126/science.1255149 PMC4369087

[39] Liu Y, Bhowmick T, Liu Y, Gao X, Mertens HDT, Svergun DI, Xiao J, Zhang Y, Wang J-H, Meijers R: Structural basis for draxin-modulated axon guidance and fasciculation by netrin-1 through DCC. Neuron 2018, 97:1261–1267.e4. 29503192 10.1016/j.neuron.2018.02.010 PMC5871715

[40] Robinson RA, Griffiths SC, van de Haar LL, Malinauskas T, van Battum EY, Zelina P, Schwab RA, Karia D, Malinauskaite L, Brignani S, : Simultaneous binding of Guidance Cues NET1 and RGM blocks extracellular NEO1 signaling. Cell 2021, 184:2103–2120.e31. 33740419 10.1016/j.cell.2021.02.045 PMC8063088

[41] Grandin M, Meier M, Delcros JG, Nikodemus D, Reuten R, Patel TR, Goldschneider D, Orriss G, Krahn N, Boussouar A, : Structural decoding of the netrin-1/UNC5 interaction and its therapeutical implications in cancers. Cancer Cell 2016, 29:173–185. 26859457 10.1016/j.ccell.2016.01.001

[42] Meier M, Gupta M, Akgül S, McDougall M, Imhof T, Nikodemus D, Reuten R, Moya-Torres A, To V, Ferens F, : The dynamic nature of netrin-1 and the structural basis for glycosaminoglycan fragment-induced filament formation. Nat Commun 2023, 14:1226. 36869049 10.1038/s41467-023-36692-w PMC9984387

[43] Priest JM, Nichols EL, Smock RG, Hopkins JB, Mendoza JL, Meijers R, Shen K, Özkan E: Structural insights into the formation of repulsive netrin guidance complexes. Sci Adv 2024, 10, eadj8083. 38363837 10.1126/sciadv.adj8083 PMC10871540

[44] Cassier PA, Navaridas R, Bellina M, Rama N, Ducarouge B, Hernandez-Vargas H, Delord J-P, Lengrand J, Paradisi A, Fattet L, : Netrin-1 blockade inhibits tumour growth and EMT features in endometrial cancer. Nature 2023, 620:409–416. 37532934 10.1038/s41586-023-06367-z PMC10412451

[45] Lengrand J, Pastushenko I, Vanuytven S, Song Y, Venet D, Sarate RM, Bellina M, Moers V, Boinet A, Sifrim A, : Pharmacological targeting of netrin-1 inhibits EMT in cancer. Nature 2023, 620:402–408. 37532929 10.1038/s41586-023-06372-2 PMC7615210

[46] Kantor DB, Chivatakarn O, Peer KL, Oster SF, Inatani M, Hansen MJ, Flanagan JG, Yamaguchi Y, Sretavan DW, Giger RJ, : Semaphorin 5A is a bifunctional axon guidance cue regulated by heparan and chondroitin sulfate proteoglycans. Neuron 2004, 44. 10.1016/j.neuron.2004.12.002 15603739

[47] Rozbesky D, Verhagen MG, Karia D, Nagy GN, Alvarez L, Robinson RA, Harlos K, Padilla-Parra S, Pasterkamp RJ, Jones EY: Structural basis of semaphorin-plexin cis interaction. EMBO J 2020, 39, e102926. 32500924 10.15252/embj.2019102926 PMC7327498

[48] Lu D, Shang G, He X, Bai X, Zhang X: Architecture of the Sema3A/PlexinA4/Neuropilin tripartite complex. Nat Commun 2021, 12:3172. 34039996 10.1038/s41467-021-23541-x PMC8155012

[49] Christie SM, Hao J, Tracy E, Buck M, Yu JS, Smith AW: Interactions between semaphorins and plexin–neuropilin receptor complexes in the membranes of live cells. J Biol Chem 2021, 297, 100965. 34270956 10.1016/j.jbc.2021.100965 PMC8350011

[50] Nagy GN, Zhao X-F, Karlsson R, Wang K, Duman R, Harlos K, El Omari K, Wagner A, Clausen H, Miller RL, : Structure and function of Semaphorin-5A glycosaminoglycan interactions. Nat Commun 2024, 15:2723. 38548715 10.1038/s41467-024-46725-7 PMC10978931

[51] Nourisanami F, Sobol M, Li Z, Horvath M, Kowalska K, Kumar A, Vlasak J, Koupilova N, Luginbuhl DJ, Luo L, : Molecular mechanisms of proteoglycan-mediated semaphorin signaling in axon guidance. Proc Natl Acad Sci U S A 2024, 121, e2402755121. 39042673 10.1073/pnas.2402755121 PMC11295036

[52] Smock RG, Meijers R: Roles of glycosaminoglycans as regulators of ligand/receptor complexes. Open Biol 2018, 8, 180026. 30282658 10.1098/rsob.180026 PMC6223220

[53] Honig B, Shapiro L: Adhesion protein structure, molecular affinities, and principles of cell-cell recognition. Cell 2020, 181:520–535. 32359436 10.1016/j.cell.2020.04.010 PMC7233459

[54] Goodman KM, Katsamba PS, Rubinstein R, Ahlsén G, Bahna F, Mannepalli S, Dan H, Sampogna RV, Shapiro L, Honig B: How clustered protocadherin binding specificity is tuned for neuronal self-/nonself-recognition. Elife 2022, 11, e72416. 35253643 10.7554/eLife.72416 PMC8901172

[55] Brasch J, Goodman KM, Noble AJ, Rapp M, Mannepalli S, Bahna F, Dandey VP, Bepler T, Berger B, Maniatis T, : Visualization of clustered protocadherin neuronal self-recognition complexes. Nature 2019, 569:280–283. 30971825 10.1038/s41586-019-1089-3 PMC6736547

[56] Guo L, Wu Y, Chang H, Zhang Z, Tang H, Yu Y, Xin L, Liu Y, He Y: Structure of cell–cell adhesion mediated by the Down syndrome cell adhesion molecule. Proc Natl Acad Sci U S A 2021, 118, e2022442118. 34531300 10.1073/pnas.2022442118 PMC8488690

[57] Cortés E, Pak JS, Özkan E: Structure and evolution of neuronal wiring receptors and ligands. Dev Dyn 2023, 252:27–60. 35727136 10.1002/dvdy.512 PMC10084454

[58] Dimitrov D, Türei D, Garrido-Rodriguez M, Burmedi PL, Nagai JS, Boys C, Ramirez Flores RO, Kim H, Szalai B, Costa IG, : Comparison of methods and resources for cell-cell communication inference from single-cell RNA-Seq data. Nat Commun 2022, 13:3224. 35680885 10.1038/s41467-022-30755-0 PMC9184522

[59] Kellermeyer R, Heydman LM, Gillis T, Mastick GS, Song M, Kidd T: Proteolytic cleavage of Slit by the Tolkin protease converts an axon repulsion cue to an axon growth cue in vivo. Development 2020, 147, dev196055. 32994163 10.1242/dev.196055 PMC7648596

